# Life‐threatening bronchospasm induced by an angiotensin‐converting enzyme inhibitor in a chronically ventilated patient: Diagnostic pitfalls and literature review

**DOI:** 10.1002/rcr2.1224

**Published:** 2023-09-20

**Authors:** Esther‐Lee Marcus, Amir Hush, Hisham Atrash, Roaia Shibli, Samuel N. Heyman

**Affiliations:** ^1^ Long‐Term Respiratory Care Division, Herzog Medical Center; Faculty of Medicine Hebrew University of Jerusalem Jerusalem Israel; ^2^ Department of Medicine Hadassah‐Hebrew University Hospital, Mt. Scopus Jerusalem Israel

**Keywords:** adverse drug reaction, angiotensin converting enzyme inhibitors, bronchospasm, chronic obstructive lung disease, mechanical ventilation

## Abstract

Cough‐ and asthma‐like symptoms are common adverse reactions to angiotensin‐converting enzyme inhibitors (ACEi). However, attributing these symptoms to the use of ACEi might be masked by clinical confounders. We report a 68‐year‐old female residing in a long‐term acute‐care facility for patients requiring prolonged invasive mechanical ventilation treated for years with ACEi. Daily reversible bouts of life‐threatening severe bronchospasm gradually developed over 6 weeks and abruptly resolved following the cessation of ACEi treatment. The late appearance of bronchospasm and the unique clinical setup of chronic invasive ventilation in a patient with smoking‐related chronic obstructive lung disease are among the principal confounders that delay the identification of the causative association between ACEi and respiratory compromise. Chronic positive pressure ventilation may also conceal small airway reactivity and obstruction, similar to auto‐positive end‐expiratory pressure (auto‐PEEP). Conceivably, angiotensin receptor blockers should be preferred over ACEi in such patients.

## Graphical abstract

A patient with chronic obstructive pulmonary disease who is managed with prolonged mechanical ventilation is described. The patient progressively developed episodes of severe bronchospasm over a few weeks. This has eventually been attributed to an angiotensin‐converting enzyme (ACE) inhibitor the patient has been treated with for years. Detailed are pitfalls in recognizing this condition, specifically in the setup of chronic invasive ventilation. Accordingly, we propose that ARBs might be preferred over ACE inhibitors in such circumstances.

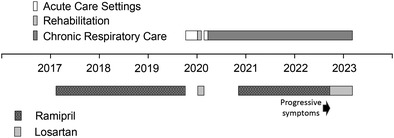

## INTRODUCTION

Introducing angiotensin‐converting‐enzyme inhibitors (ACEi) and angiotensin II‐AT1‐receptor blockers (ARBs) attenuates hypertension and provides substantial cardio‐renal protection with reduced mortality.^1^, ^2^, ^3^, ^4^ This reflects the suppression of the ‘pressor’ renin/angII/AT1R/aldosterone axis, which, in addition to vasoconstriction, stimulates inflammation, fibrosis, hypercoagulation, and apoptosis, generating hypertension and progressive vascular, myocardial, and renal injury. Blocking this harmful axis diverts the angiotensinogen‐derived system towards the ‘depressor’ ACE‐2/ang‐(1–7)/MasR axis that counterbalances the injurious pressor axis, exerting vasodilation and inhibiting inflammation, hypercoagulation, apoptosis, and fibrosis.^5^ Importantly, angiotensin‐converting enzyme (ACE), a protease that converts angiotensin I to angiotensin II, also degrades bradykinin. The consequent increase in bradykinin and substance P bioavailability is likely responsible for the major adverse reactions to ACEi, namely cough, bronchospasm, and angioedema.^6^, ^7^ Cough is the most common adverse response. A large controlled study revealed that its prevalence in patients on ACEi was 12.3%, compared with 2.7% among controls. Bronchospasm is less common (5.5% vs. 2.3%), unrelated to a history of bronchospasm, and is not dose‐dependent.^8^ Literature review suggested that reactive airway disease does not affect the propensity to bronchospasm on ACEi.^9^ However, Morales et al. reported that among 642,336 new users of ACEi, asthma increased the likelihood of switching from ACEi to ARBs by 16%, underscoring the potential of this class of drugs to exacerbate small airway disease.^10^ Furthermore, forced expiratory volume at 1 s and forced vital capacity were significantly lower in patients with hypertension treated with ACEi than in patients on ARB.^11^ Other features of bradykinin‐mediated reactions are far less common, with angioedema reported in up to 0.7% of patients treated with an ACEi.^7^ ACEi likely intensify the risk of anaphylactic response to other substances, such as iodine‐containing contrast media.^12^

The late onset of these adverse reactions and lack of awareness often lead to late recognition of their link to the use of ACEi. For instance, on average, it took 293.2 days to conclude that ACEi administration is responsible for a persistent cough in 446 spontaneous notifications by primary care physicians registered in the French pharmacovigilance database. When reported (*n* = 179), the average time from ACEI introduction to cough onset was 156.8 days.^13^ A very delayed presentation following the initiation of ACEi has also been reported with anaphylactic reactions,^14^ often following the concomitant administration of non‐steroidal anti‐inflammatory agents (NSAIDs).^15^ Regarding bronchospasm, the onset symptoms are usually within the first couple of weeks after initiating ACEi treatment. However, late‐onset may also occur,^16^ promoting overlooking possible linkages.

From these perspectives, an adverse response to ACEi might be overlooked in patients with an already known airway disease, especially if drug‐related symptoms develop long after initiating treatment. Herein, we report and discuss the challenges leading to delayed identification of ACEi‐related bouts of severe bronchospasm in a patient managed with prolonged invasive mechanical ventilation who has been treated with an ACEi for years.

## CASE REPORT

Progressive dyspnea developed in a 68‐year‐old female residing in a long‐term acute care facility for patients requiring prolonged invasive mechanical ventilation. The patient is morbidly obese, hypertensive, and has quit smoking. She has non‐insulin‐dependent diabetes and chronic obstructive pulmonary disease (COPD). She required oxygen supplementation for a decade and was treated with nocturnal BIPAP, bronchodilators, antihypertensive medications, and diuretics. The patient was functionally independent but complained of dyspnea on exertion related to COPD and hypertensive heart disease. Thirty months before respiratory deterioration, the patient underwent partial small bowel resection following an incarcerated ventral hernia. A protracted convalescence period was required before weaning off the ventilator. Bouts of dyspnea and bronchospasm were managed using bronchodilators, steroids, and diuretics. Unfortunately, a successful rehabilitation course over 3 months was truncated by relapsing respiratory failure, attributed to pneumonia and sepsis, and subsequently, the need for intubation and mechanical ventilation. Failed weaning attempts resulted in tracheostomy and prolonged mechanical ventilation. For 26 months, the patient was stabilized at our institution on pressure‐support synchronized intermittent mandatory ventilation. The patient was mobile and partially independent in activities of daily living. Occasional rare episodes of COPD exacerbation were managed with antibiotics, inhaled steroids, bronchodilators, and short courses of systemic steroids. Uncontrolled diabetes requires the administration of insulin and metformin at the cost of 18 kg of weight gain. However, reversible bouts of severe respiratory distress with bronchospasm eventually developed over 6 weeks at an increasing rate. Dyspnea attacks were characterized by an abrupt onset with labored and prolonged expiration associated with tachycardia, pallor, and cold diaphoresis. Blood pressure increments of up to 230/140 mmHg were observed, with a prominent pulsus paradoxus. The tidal volumes on the ventilator declined to 250 mL, with end‐tidal carbon dioxide (CO_2_) reaching 80 mmHg and oxygen saturation falling to 70% with 30% inspired oxygen.

Features of pulmonary infection did not precede the increase in the frequency and severity of bronchospasm. Orthopnea between attacks was absent, as was chest pain and foamy or bloody sputum. The patient denied having calf tenderness or leg swelling. Inspiration was unlabored, and suction attempts excluded upper airway obstruction or excessive sputum. Physical examination during the attacks revealed prolonged and labored expiration and diffuse expiratory wheezing in all lung fields. No features of heart failure or deep vein thrombosis were observed. ECG recordings during the attacks revealed sinus tachycardia without ischemic changes or indicators of pulmonary hypertension. Chest radiography excluded overt pulmonary congestion, consolidation, or other anomalies.

Agitation and fighting the ventilator required manual ventilation on a few occasions. Ipratropium bromide, Fluticasone, and Salbutamol were also administered. Systemic steroids were administered, and morphine was occasionally required to attenuate distress and respiratory workload. Intravenous nitrates and furosemide were administered without an overt or rapid clinical response. The attacks slowly abated over a few hours but recurred daily. Intensification of the antihypertensive regimen resulted in near‐normal blood pressure between exacerbations. However, worsening attacks became more frequent, occurring 2–3 times daily. Diabetes became uncontrolled, as did bulimia, necessitating increasing doses of insulin.

Adverse drug reactions were considered, with the clinical exclusion of cardiac asthma, thromboembolism, carcinoid syndrome, and pheochromocytoma. The patient did not receive NSAIDs or beta‐blocking agents; however, hypertension was managed with ramipril.

**FIGURE 1:**
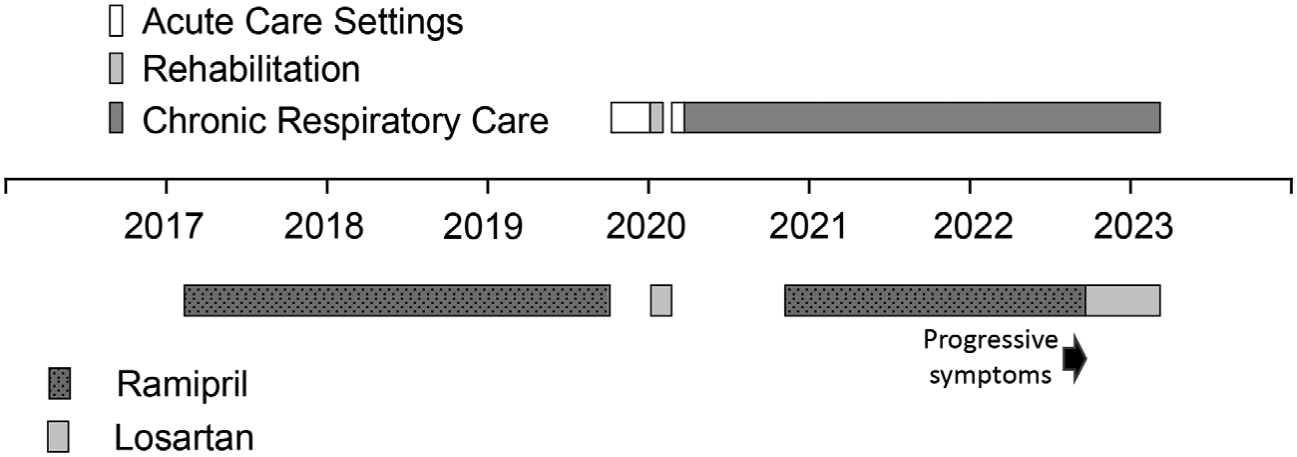
The patient's time scale illustrating medications and allocation. Management of hypertension with ramipril and losartan are shown at the bottom. Illustrated on top is the allocation to different medical facilities.

A retrospective evaluation revealed that treatment with ramipril (2.5 mg/day), initiated by the family practitioner 5.5 years earlier (Figure 1), was halted for nearly a year during and following the patient's emergency hospitalizations, transiently substituted by losartan. However, ramipril was resumed and continuously administered for 22 months before the recent clinical deterioration.

Episodes of severe dyspnea and respiratory failure abruptly stopped the day ramipril was replaced with losartan, and the patient remained free of bronchospasms for the following 8 months.

## DISCUSSION

Iatrogenesis is a major cause of morbidity and mortality worldwide. Over 20 years ago, iatrogenic causes were responsible for 225,000 deaths in the US, the third leading cause of death after heart disease and cancer.^17^ Globally, the costs associated with medication errors have been estimated at USD 42 billion annually.^18^ Adverse drug reactions have been reported in about 20% of patients in emergency rooms.^19^ In a systematic review of studies addressing adverse drug reactions‐induced hospital admissions in patients over 60 years of age, the overall average percentage of hospital admissions related to adverse drug reactions was 8.7% among all admissions.^20^ Iatrogenic illness is a major cause of additional in‐hospital life‐threatening complications and death.^21^, ^22^ The described sequence of events illustrates a near‐fatal example of overlooked iatrogenesis.

One may ask why it took so long to identify the cause of the progressive dyspnea in our patient. The setup of mechanical ventilation is likely a principal confounder; cough and occasional dyspnea are common complaints in such patients and are related to irritation of the tracheal mucosa, secretions, mucus plugs and microaspirations. This setup is far from that of new‐onset respiratory symptoms, principally cough, in non‐ventilated individuals. Furthermore, a history of smoking‐related COPD, CO_2_ retention, diastolic cardiac dysfunction, and morbid obesity with restrictive lung disease are likely additional major confounders. Positive pressure ventilation also masked the features of bronchiolar constriction to some extent, alleviating small airway obstruction. Prolonged treatment with ACEi, extending approximately 2 years before respiratory compromise developed, and the lack of other features of anaphylaxis, such as angioedema, very likely led to overlooking their plausible role in the clinical presentation. Along this line, we have occasionally encountered patients chronically treated with ACEi who developed protracted cough following intercurrent respiratory tract infection, with complaints resolving only after cessation of ACEi. Thus, prolonged and uneventful treatment with ACEi does not exclude their potential role in new‐onset cough or bronchospasm, and its late occurrence has indeed been previously reported.^13^, ^16^ The absence of aggravated cough, a prominent symptom of ACEi‐related adverse responses, likely served as another confounder in our patient. We propose that positive‐pressure ventilation eliminates the need to maintain small airway patency using cough‐ or pursed‐lip‐generated positive end‐expiratory pressure (auto‐PEEP). Furthermore, as congestive heart failure predisposes to ACEi‐related symptomatic bronchospasm,^9^ possibly, positive pressure mechanical ventilation concealed subclinical heart failure with preserved ejection fraction (HFpEF) in our patient.

Finally, the lack of continuity in medical care among patients moving from one medical care system to another is an additional plausible factor in overlooking ACEi‐mediated adverse effects. The transfer of patients between primary care and ambulatory or non‐ambulatory medical systems or between critical care units and regular wards forms a frail link in the chain of securing the continuity of appropriate medical assessment.^23^, ^24^ Although lists of medications and known allergies are mandatorily provided during the transition of care, the rationale for therapeutic decisions is often not detailed in discharge or referral letters. It is tempting to assume that treatment with ACEi in our patient was not resumed during prolonged postsurgical critical care management because of the emergence of adverse respiratory effects. However, this consideration has not been outlined during relocation. Underscoring this concern in the discharge summaries might have elucidated previous adverse reactions to ACEi that could have been prevented.

In conclusion, our case report illustrates that since cough and bronchospasm are the principal respiratory adverse reactions to ACEi, ARBs may be preferred over ACEi in patients with COPD, especially those on invasive mechanical ventilation, to avoid confusing drug‐induced symptoms with those related to the underlying respiratory tract disorder.

## AUTHOR CONTRIBUTIONS

All authors treated the patient. Amir Hush, Hisham Atrash, and Roaia Shibli contributed to data collection. Roaia Shibli, Esther‐Lee Marcus, and Samuel N. Heyman performed the literature search. Esther‐Lee Marcus and Samuel N. Heyman prepared the manuscript. All authors have read and approved the final version of the manuscript.

## CONFLICT OF INTEREST STATEMENT

None declared.

## ETHICS STATEMENT

The authors declare that appropriate written informed consent was obtained for the publication of this manuscript and accompanying images.

## Data Availability

Data available on request due to privacy/ethical restrictions.
